# The captivating role of calcium in plant-microbe interaction

**DOI:** 10.3389/fpls.2023.1138252

**Published:** 2023-03-03

**Authors:** Anirban Bhar, Amrita Chakraborty, Amit Roy

**Affiliations:** ^1^ Post Graduate Department of Botany, Ramakrishna Mission Vivekananda Centenary College, Kolkata, India; ^2^ Faculty of Forestry and Wood Sciences, Czech University of Life Sciences, Prague, Czech Republic

**Keywords:** calcium signalling, calcium-dependent proteins, defence signalling, plant-microbe interaction, ROS, biotic stress

## Abstract

Plant immune response is fascinating due to the complete absence of a humoral system. The adaptive immune response in plants relies on the intracellular orchestration of signalling molecules or intermediates associated with transcriptional reprogramming. Plant disease response phenomena largely depend on pathogen recognition, signal perception, and intracellular signal transduction. The pathogens possess specific pathogen-associated molecular patterns (PAMP) or microbe-associated molecular patterns (MAMP), which are first identified by pattern recognition receptors (PRRs) of host plants for successful infection. After successful pathogen recognition, the defence response is initiated within plants. The first line of non-specific defence response is called PAMP-triggered immunity (PTI), followed by the specific robust signalling is called effector-triggered immunity (ETI). Calcium plays a crucial role in both PTI and ETI. The biphasic induction of reactive oxygen species (ROS) is inevitable in any plant-microbe interaction. Calcium ions play crucial roles in the initial oxidative burst and ROS induction. Different pathogens can induce calcium accumulation in the cytosol ([Ca^2+^]_Cyt_), called calcium signatures. These calcium signatures further control the diverse defence-responsive proteins in the intracellular milieu. These calcium signatures then activate calcium-dependent protein kinases (CDPKs), calcium calmodulins (CaMs), calcineurin B-like proteins (CBLs), etc., to impart intricate defence signalling within the cell. Decoding this calcium ionic map is imperative to unveil any plant microbe interplay and modulate defence-responsive pathways. Hence, the present review is unique in developing concepts of calcium signature in plants and their subsequent decoding mechanism. This review also intends to articulate early sensing of calcium oscillation, signalling events, and comprehensive mechanistic roles of calcium within plants during pathogenic ingression. This will accumulate and summarize the exciting roles of calcium ions in plant immunity and provide the foundation for future research.

## Highlights

• Plant-microbe interaction is the pioneer field of study in plant science as it is directly related to crop productivity and global food security.
• Interesting findings summarizing plant immunity are popping up each day, developing new ideas and concepts.
• Calcium signalling is the paramount event in any plant biotic ingression.
• The development of calcium signature and decoding of these signalling interfaces is the intricate signal transduction machinery.
• The concept of “calcium signature” and pathogen-specific concentration gradient is necessary to understand the signalling events properly.
• The present review not only tries to comprehend the “calcium signature” concept but also elaborately focuses on the different levels of decoding machinery of this signature in plants.
• More interestingly, the crosstalk of different signalling pathways is elaborated in this review which is essential to develop inherent effective resistance response. ROS and calcium signalling is intricately associated with one another. The emerging signalling overlap between MAPK and calcium is documented in this study also.

## 1 Introduction

Plants cells are the storehouse of different ions in an aqueous milieu. The chemistry of life depends on the dynamic changes of this intracellular anion and cation level. The fundamental functionality of the cell depends primarily upon the proton, H^+^, and other cations. The cellular metabolism and associated enzyme action solely depend upon the specific pH of the cell (Kader and Lindberg, 2010). Along with the H^+^ and other cations, calcium (Ca^2+^) is an enormously crucial bivalent cation with varying plant utilities. These Ca^2+^ ions have structural, nutritional, and stress-inductive functions (Thor, 2019). The cell wall integrity incessantly depends upon the Ca^2+^ for cross-linking, thus providing principal storage of Ca^2+^ ions in plants (Hepler and Winship, 2010). Besides, Ca^2+^ can also be stored in mitochondria, chloroplast, and vacuoles. The apoplast also plays a pivotal role in the calcium cycle in plants (He et al., 2021). The primary source of intracellular calcium is the soil, and unlike the most available cation, it is challenging to prevail against calcium deficiency in soil (White and Broadley, 2003). Calcium uptake difficulties may occur due to several greenhouse conditions, temperature stress, drought, chelation, etc. The Ca^2+^ deficiency, although rare but detrimental when it appears. The symptoms include stunted growth, black spots, and an unusually bushy appearance. Excess fertilisation may also cause Ca^2+^ deficiency and is usually identified in young leaf tips, characterised by the “bull-whipping” or “buggy-whipping” phenotype in maize (Wang et al., 2020). Besides nutritional and structural roles, Ca^2+^ is an essential secondary messenger in cellular signalling events. Ca^2+^ perturbations are inevitable in any biotic or abiotic stress response (Saijo and Loo, 2020; Yadav et al., 2022). In response to stress, Ca^2+^ concentration is spiked within the cell cytosol. The concentration of Ca^2+^ within cell cytosol is maintained by different classes of calcium influx and efflux protein (Bose et al., 2011). The Ca^2+^ ions thus accumulated in the cytosol in any plant-microbe interaction may directly control the cellular redox homeostasis or act as a second messenger to regulate calcium ion-dependent gene expression (CDGE) (Cao et al., 2022). Different cellular and stress responses can generate unique and precise calcium spikes in cytosol called “calcium signatures” (McAinsh and Pittman, 2009). The cell wall, apoplast, vacuoles, and different cell organelles participate in this calcium flow. This collective Ca^2+^ concentration is then sensed by different calcium-sensing proteins, e.g., calcium-dependent protein kinase (CDPKs), calcium calmodulins (CaMs), calcineurin B-like proteins (CBLs), calreticulin, etc. (Gao et al., 2019). The specific calcium signature has then become decoded by these calcium sensors and transduced into specific downstream signalling. The reactive oxygen species (ROS) generation is directly associated with intracellular Ca^2+^ in plants (Marcec et al., 2019; Singh et al., 2022). Further, CDPKs targeted different defence-responsive proteins to impart resistance response. Integrating other signalling modules and hormonal signalling is also connected with the calcium signalling pathway (Trotta et al., 2014). The present review focus on comprehensive calcium signalling in plant-microbe interaction, in which the “calcium signature” concept, sensing, and decoding mechanism of the calcium concentration pool is also elaborated. The obvious imbrication of Ca^2+^-ROS signalling and emerging cross-talk between the Ca^2+^-MAP kinase (MAPK) cascade is also documented in connection to biotic ingression.

## 2 Plant-microbe interaction: a needle in a haystack

The absence of humoral immunity in plants and sole dependence on adaptive immunity is governed by myriad pathogen-specific proteins and their cognate receptors in host cells. Plant immunity is best described by the intellectual “Zig-Zag model” proposed by Jones and Dangl (Jones and Dangl, 2006). This model gets enormous popularity (>350K accesses and > 12000 citations as of December 2022) because it is the first to describe plant immune response comprehensively. According to this model, the pathogen bears some signature chemical compounds, e.g., flg22 (flagellin protein), elf18 (N terminal elongation factor Tu) from the bacterial pathogen, chitin from the fungal pathogens those acts as the recognition molecules for the pathogen, called pathogen-associated molecular pattern (PAMP) or microbe-associated molecular pattern (MAMP). These PAMPs/MAMPs have cognate receptors in the host cell surface, e.g., FLS2 for the flg22, called pattern recognition receptors (PRRs). PAMP-PRR interaction leads to the first phase of immune response in the “Zig-zag” system, which is characterised by a transient increase in reactive oxygen species (ROS), Ca^2+^ influx, and activation of some transcription modulators (Gupta et al., 2013). This phenomenon is called PAMP-triggered immunity (PTI). The second phase of plant immunity is more robust and specific to the pathogen types than the more generalised PTI. During ETI (effector-triggered immunity), pathogens released specific toxins identified and detoxified by the different classes of receptors and resistance genes (R). The effector-binding proteins are members of highly diversified nucleotide-binding leucine-rich repeat receptors (NLRs) in plants. This immune response may further develop priming or immunogenic memory by diverse modes of action (Bhar et al., 2021). The discovery of the different classes of PAMPs/MAMPs, PRRs, NLR, etc., and the gradual enumeration of their mode of action progressively faded proper distinction between PTI and ETI (Thomma et al., 2011). The leucine-rich repeats (LRR) proteins present in the host cell surface are of two types, receptor-like proteins, RLPs, and receptor-like kinases, RLKs. The LRR usually interacts with extracellular immunogenic patterns (ExIPs) (previously discussed, PAMPs/MAMPs and any molecules capable of activating the autophosphorylation module of the LRRs) and recruit LRR-RLK-BAK1. In contrast, RLPs interact with the suppressor of BIR1‐1 (SOBIR1) to instigate downstream immunogenic pathways (Van Der Burgh et al., 2019). Hence, recently it has been argued that plant immunity is better classified as extracellularly triggered immunity (ExTI) and intracellularly triggered immunity (ITI) (van der Burgh and Joosten, 2019). The primary immune response is then radiating into a multitude of signal transduction and intracellular cross-talk to develop sustainable resistance responses in plants.

The above section describes the general biphasic induction of plant immunity and their different components as elaborated by the classical “zig-zag model”. It also concludes the transition of PTI and ETI towards ExTI and ITI in the modern era of plant immunity.

## 3 The concept of “calcium signature”

**Figure 1:**
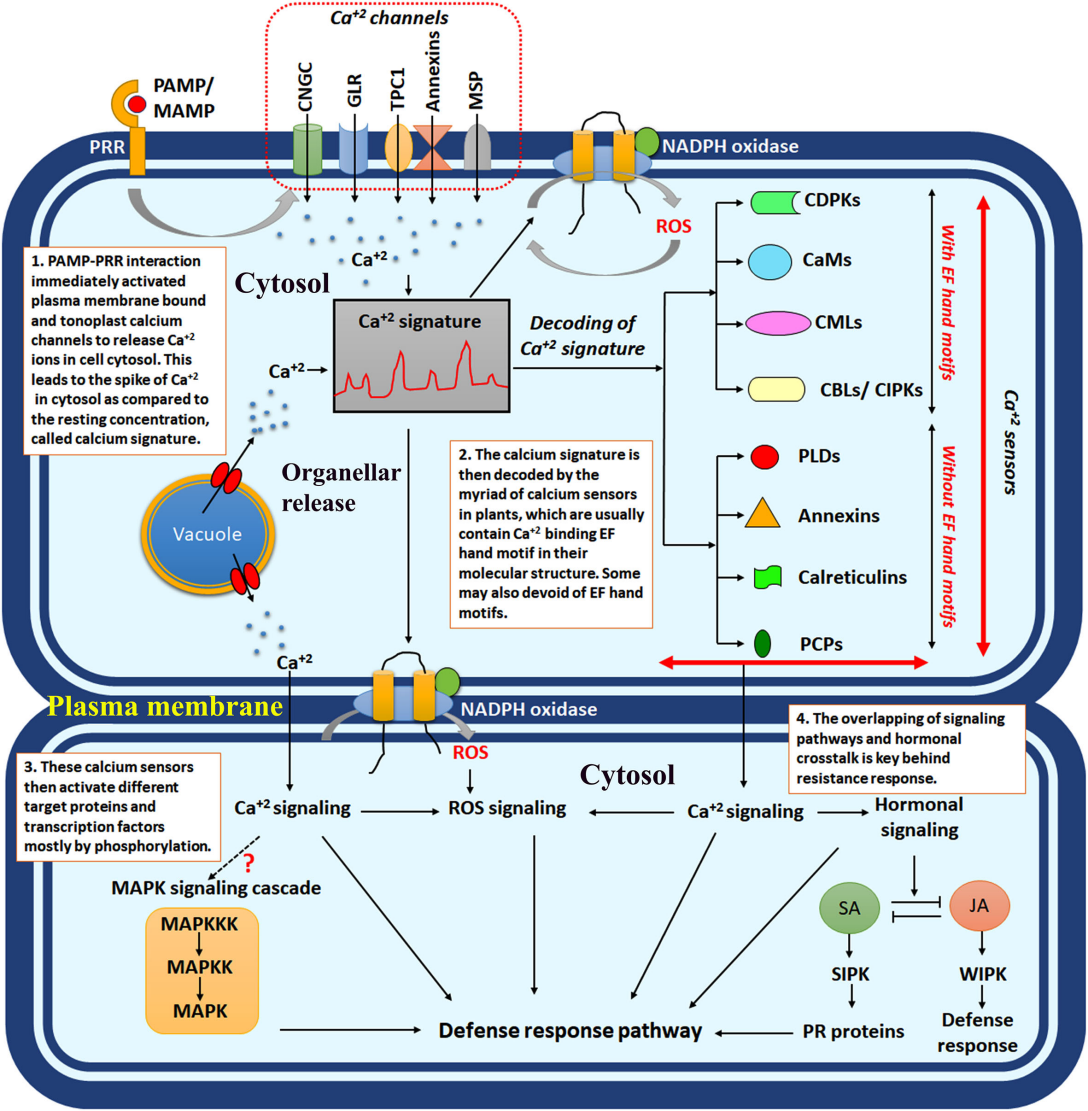
The schematic diagram showing intracellular calcium signalling in response to plant-microbe interaction. PAMP/MAMP, pathogen-associated molecular pattern/microbe-associated molecular pattern; PRR, pattern recognition receptors; CNGCs, cyclic nucleotide-gated channels; GLR, ionotropic glutamate receptors; TPC1, two-pore channel 1; MSP, mechanosensitive protein channels; CDPKs, calcium-dependent protein kinases; CaMs, calcium calmodulins; CMLs, CaM like proteins; CBLs, calcineurin B like proteins; SA, salicylic acid; JA, jasmonic acid; SIPK, salicylic acid induced protein kinase; WIPK, wound-induced protein kinase; ROS, reactive oxygen species.

**Figure 2:**
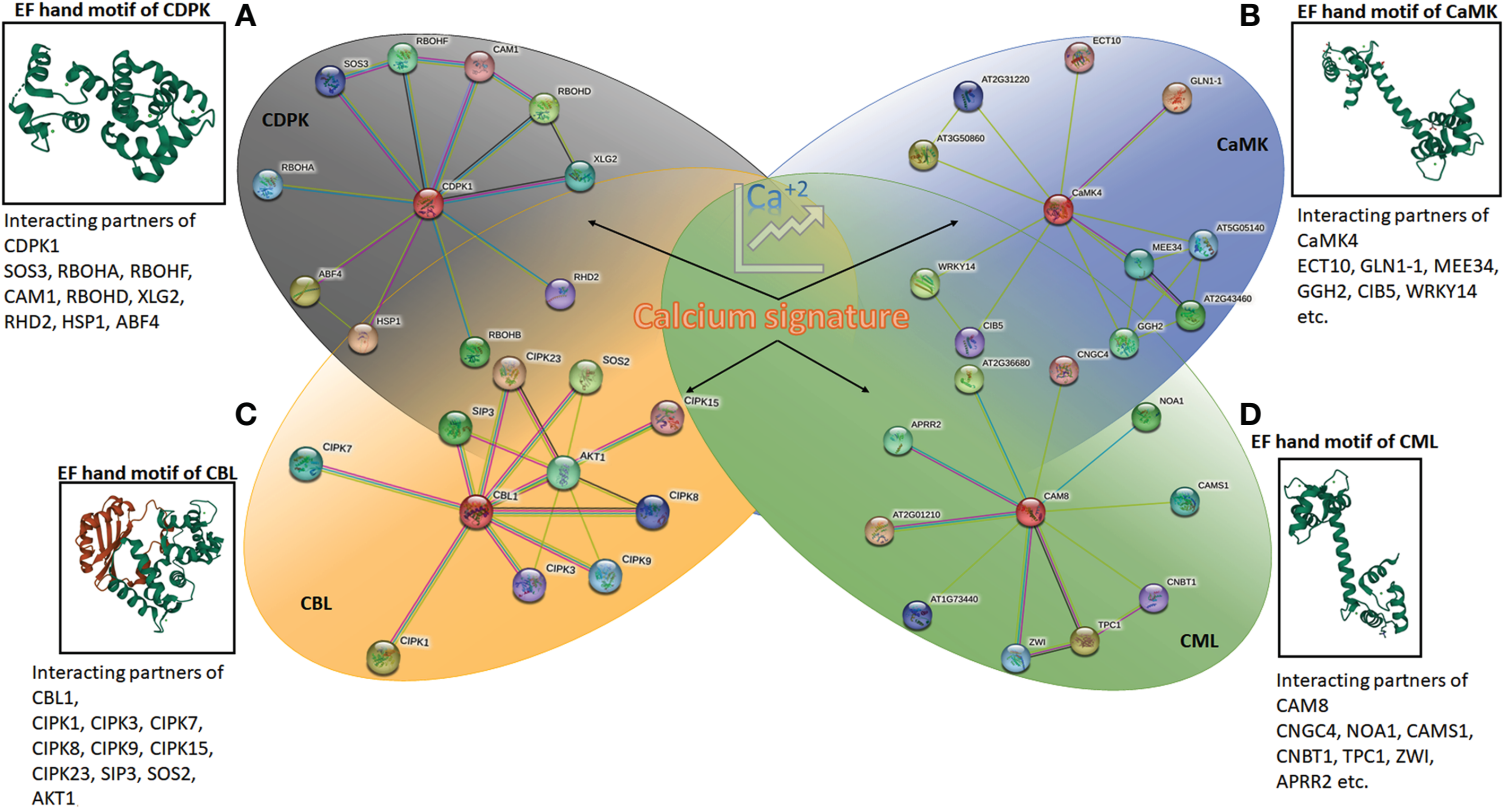
The protein-protein interaction network generated using STRING (version 11.5) online server using *Arabidopsis* CDPK1, CaMK4, CAM8 and CBL1. The details of interacting partners are summarised in **Supplementary Excel 1 and 2**. The molecular structure of calcium-binding EF-hand motifs is also depicted from different reported protein data bank (PDB) structures. **(A)** CDPK of *Arabidopsis* (2AAO) reported by Chandran et al. (2006); **(B)** CaM7 (5A2H) of *Arabidopsis thaliana* reported by Kumar et al., (2016); **(C)** SOS2 interacting with SOS3 (2EHB), CBL of *Arabidopsis thaliana* reported by Sánchez-Barrena et al. (2007); and **(D)** calmodulin, CML (1UP5) from chicken reported by Rupp et al. (1996).

Calcium is an important signalling intermediate in plants. This simple bivalent cation has enormous utility and function within plants, from developmental purpose to stress response (DeFalco et al., 2023). The calcium production, its cytosolic concentration [Ca^2+^] _Cyt_, and subsequent decoding mechanism determine the fate of the signal transduction. The [Ca^2+^]_Cyt_ and the judicial transporter system control the entire network. The specific cytosolic or organellar calcium concentration may instigate distinct sets of signalling intermediates to perform specific functions. Such Ca^2+^ concentration is called “calcium signature”. In the polarised cell growth in root hairs and pollen tubes, the calcium channels are localised in the tip cells and activate the CNGC (cyclic nucleotide-gated channels) and GLR (glutamate receptors) class of calcium channels (Tian et al., 2020). The six members, mainly CNGC 7, CNGC 8, CNGC 9, CNGC 10, CNGC 16 & CNGC 18, are highly expressed in the tip cells in *Arabidopsis* (Frietsch et al., 2007; Tunc-Ozdemir et al., 2013a; Tunc-Ozdemir et al., 2013b). Similarly, plant-microbe interaction causes Ca^2+^ spike and oscillation in the cytosol, whereas nuclear spike is observed in symbiotic interactions (Tian et al., 2020). Prolonged Ca^2+^ influx and intermediate oscillation are observed in systemic response in plants (Aldon et al., 2018). Instead, recently, it was observed that in response to flg22, systemic tissue does not impose rapid induction of calcium oscillation but rather calcium-dependent downstream signalling instigated in *Arabidopsis thaliana* (Eichstädt et al., 2021). Uncontrolled production of calcium, overactivation of calcium channels, or autoregulated calcium influx may negatively affect the plant immune system and cause inappropriate defence response. The negative role of calcium due to overproduction is mainly controlled by AtCPK28, CNGC 2, and CaM-binding transcriptional factor 3 (CaMTA3) in the case of *Arabidopsis thaliana* (Yuan et al., 2017). It was evident that the BONZAI1 protein interacts with autoinhibitory domains of autoinhibited calcium ATPase10 (ACA10) and ACA8 of the plasma membrane and regulates the cytosolic calcium signatures. The *aca10* and *bon1* mutants exhibited autoimmune phenotype in *Arabidopsis thaliana*, and constant increase in cytosolic calcium leads to impaired stomatal closure in response to pathogens (Yang et al., 2017). The calcium concentration in the shoot tissue oscillates between 0.1% to 5% of the total dry weight of the plants (Jose, 2023). During the uninduced situation, the Ca^2+^ concentration in the cytosol remains in a steady state of 0.1μM, which is achieved by the diverse calcium channels, e.g., Ca^2+^-ATPases and H^+^/Ca^2+^ antiporters (Thor, 2019). During the stress response, this Ca^2+^ concertation gradually increases within the cytosol by coming down the concentration gradient from the apoplast or vacuolar storage. The apoplastic Ca^2+^ concentration usually remains 10000-fold more than that of cytosol (Nomura and Shiina, 2014). Alternatively, chloroplast, another calcium storehouse, maintains steady-state Ca^2+^ concentrations. The “resting concentration” of Ca^2+^ within stroma is 150nM, whereas the same in the thylakoid lumen is 15mM, which means the calcium is sequestered within the lumen in uninduced condition (Johnson et al., 1995; Nomura and Shiina, 2014). The spike of the intracellular Ca^2+^ levels was observed in response to any stress and biotic ingression. This precise balance is governed by well-orchestrated transporters and a Ca^2+^ buffering system (Demidchik et al., 2018) (**Figures 1**, **2**).

The above section describes the specific concentrations of Ca^2+^ within the plant cell in response to specific stress. This specific concentration of Ca^2+^ is designated as the “calcium signature”. The intracellular “calcium signature” has been achieved by coordinating the influx/efflux channels of different intracellular organelles. They possess extraordinary roles in plant development and stress response. The opposing roles of Ca^2+^ have also been elaborated.

## 4 The role of calcium in plant-microbe interaction

### 4.1 Calcium channel

In plants, Ca^2+^ is exchanged by different channel proteins, e.g., cyclic nucleotide-gated channels (CNGCs), ionotropic glutamate receptors (GLR), two-pore channel 1 (TPC1), annexins, and several types of mechanosensitive channels. In *Arabidopsis*, about 150 cation transporters have been reported to date (Mäser et al., 2001), among them 20 are the CNGC class of Ca^2+^ transporters (Tian et al., 2020). These class of Ca^2+^ transporters are usually located to plasma membrane which senses intracellular levels of cyclic nucleotides monophosphates (cNMPs), e.g., adenosine 3′,5′-cyclic monophosphate (cAMP) and guanosine 3′,5′-cyclic monophosphate (cGMP) and controls the Ca^2+^ levels to transduce different signalling events. In this function, phosphodiesterase (PDEs) enzymes play a crucial role in regulating intracellular cNMP levels (Duszyn et al., 2019). As discussed, PAMP-PRR interaction is the hallmark of any plant-microbe interplay. CNGC2 and CNGC4 were known to induce ETI response by activating ROS generation in *Arabidopsis* in response to flg22, the universal PAMP of bacterial pathogens (Tian et al., 2020). The hypersensitive response (HR) mediated cell death is observed as a part of the ETI response in *Arabidopsis* and is regulated by CNGC2. CNGC2/defence, no death1 (DND1) was reported to regulate intracellular nitric oxide levels that controls the defense response. The *cngc2/dnd1* mutants showed no accumulation of Ca^2+^ and thus exhibited no HR (Ali et al., 2007). Complementation and mutant analysis demonstrated that CNGC11 and CNGC12 are also involved in programmed cell death in response to pathogenesis in a caspase (VPE, vacuolar processing enzyme) dependent nature (Urquhart et al., 2007). It has recently been observed that CNGC20 also plays a critical role in plant immunity, interacting with CNGC19 and BOTRYTIS INDUCED KINASE1 (BIK1). The ENHANCED DISEASE SUSCEPTIBLITY1 (EDS1) controls ETI; the *eds1* mutants restore disease resistance when CNGC20 is overexpressed (Zhao et al., 2021).

The GLR-type Ca^2+^ receptors are also common in plants which share structural similarities with animal ionotropic glutamate receptors. In *Arabidopsis*, 20 GLR-type Ca^2+^ transporters have been reported (Lam et al., 1998; Lacombe et al., 2001), and most of them are known to have developmental functions. It was observed that GLR3.1 and GLR3.5 directly control Ca^2+^ uptake in cells and regulate ROS production when activated with a physiological concentration of L-methionine (L-Met) (Kong et al., 2016). H^+^/amino acid symporters maintain the optimal glutamate concentration required for the action of GLR3.3. Along with the glutamate, there are six amino acids, e.g., glycine, alanine, serine, asparagine, and cysteine, as well as the tripeptide glutathione (γ-glutamyl-cysteinyl-Gly), was also found to be potent agonists to GLR3.3; which indicates interaction of GLR3.3 and amino acids in the rhizosphere region to control intracellular Ca^2+^ concentrations (Qi et al., 2006). Although most of the functions of GLR transporters are restricted to developmental functions, genome-wide functional studies have recently reported some of the soybean GLRs in response to stress (Jia et al., 2022). In an independent study, it was observed that exogenous treatment of Glu can induce an immune response in *Arabidopsis* by activating PTI-responsive genes (BIK1, BKK1, BAK1, CERK1, PBL1, etc.), LYSIN-MOTIF RECEPTOR-LIKE KINASE 5 (LYK5) (which is a chitin receptor) and salicylic acid biosynthetic genes (SID2) (Goto et al., 2020).

The two-pore channel 1 (TPC1) is the dimeric membrane channel predominantly located in the vacuole of plants and is responsible for maintaining vacuolar Ca^2+^ levels (Demidchik et al., 2018). This group of Ca^2+^ channels is called the “slow vacuolar” (SV) channel, and one single member of this gene is found in *Arabidopsis* (Peiter et al., 2005). Not much work has been done on TPC1 concerning plant-microbe interaction, but their role in Ca^2+^ transport across tonoplast membranes has been confirmed in *Arabidopsis* through a cross-species complementation study (Dadacz-Narloch et al., 2013). TPC1 maintained the optimal cytosolic Ca^2+^ by sequestering excess calcium in vacuoles to prevent ROS-mediated HR and programmed cell death in plant cells.

Annexins are the large group of calcium-sensing proteins in the cytosol of eukaryotic cells and are mainly absent in prokaryotes. The first discovered annexin is called “synexin” (now called annexin A7) in humans (Mirsaeidi et al., 2016). Although plant annexins differ significantly from animal annexins, they are involved in Ca^2+^-dependent association with plasma membrane phospholipids (Demidchik et al., 2018). The eight annexin-encoding genes had been reported in *Arabidopsis*, among them annexin 1 (ANN 1), which transported Ca^2+^ in a ROS-dependent manner (Davies, 2014). The MtANN1 was upregulated in the early time points of infection in *Medicago truncatula* against root-inhabiting bacteria *Rhizobium meliloti* infection (de Carvalho Niebel et al., 1998). The role of PsANN4 and PsANN8 in symbiotic interaction was also recently established in Peas (*Pisum sativum*) (Pavlova et al., 2021). The role of annexins in symbiotic interaction was further confirmed in common beans (*Phaseolus* sp), where downregulation of *Phaseolus* annexin genes was associated with impaired infection and nodulation (Carrasco-Castilla et al., 2018). Although out of the scope of this article, recently, the role of annexins in plant-insect/plant-parasitic interaction was also evident where interestingly, annexins play a pivotal role in Ca^2+^ mediated signalling and defence response (Gupta and Roy, 2021; Hundacker et al., 2022; Onofre et al., 2022).

Membrane tension and osmolarity-dependent Ca^2+^ transport have also taken place by some mechanosensitive ion channels. These channels are primarily classified into mechanosensitive-like channels (MSLs), mechanosensitive ‘Mid1-complementing activity’ channels (MCAs), and mechanosensitive piezo channels (Hamilton et al., 2015). These channel proteins have diverse structural variability, e.g., mitochondria-chloroplast MSLs were reported to have five transmembrane domains assembled in heptamers, whereas plasma membrane MSLs have six transmembrane domains assembled in tetramers (Hamilton and Haswell, 2017). MSL 10 demonstrated resistance to bacterial pathogen, *Pseudomonas syringae* in *Arabidopsis thaliana* by inducing controlled ROS and activating SID2, PAD4, EDS1, and NDR1 (Basu et al., 2022) (**Figure 1** and **Table 1**).

### 4.2 Calcium sensors: The decoding of calcium signature and downstream signalling

#### 4.2.1 EF hand motifs

Different classes of Ca^2+^ sensor proteins sense the calcium influx or spike within the cell cytosol. These proteins sense the Ca^2+^ ion through a helix-loop-helix domain called EF-hand motifs. This structure was first reported in “parvalbumin”, a calcium-binding protein found in the muscle cells of human beings (Kretsinger and Nockolds, 1973). As the charged molecule, Ca^2+^ interacts with the negatively charged aspartate and glutamate residues in EF-hand motifs (Falke et al., 1994). The seven ligands bind the Ca^2+^ ions in pentagonal bipyramid geometry within the EF-hand motif (La Verde et al., 2018). The proteins consist of either D-x-D motifs in each of their EF-hands or D-x3-D motifs in the first and second hand, which bind the calcium (Mohanta et al., 2019). Among the four major classes of calcium sensors in plants, calcium-dependent protein kinases (CDPKs) and calcium calmodulins (CaMs) contain D-x-D motifs, whereas, CaM-like proteins (CMLs) hold D-x3-D motifs. The calcineurin B-like proteins (CBLs) are the only class of plant Ca^2+^ sensors bearing three EF-hands with characteristics V-F-H-P-N, D/E-x-D, and D-x-E-E motifs (Mohanta et al., 2015) (**Figure 2**).

#### 4.2.2 Calcium-dependent protein kinases

CDPKs are the most diverse group of calcium sensors present in plants. More explicitly, CDPKs can further be classified into five different types, (i) Ca^2+^ dependent protein kinases, which are technically CaM independent (CDPKs); (ii) CDPK-related protein kinases (CRKs); (iii) CaM dependent protein kinases (CaMKs); (iv) Ca^2+^/CaM-dependent protein kinases (CCaMK); (v) SOS3/CBL interacting protein kinases (SIPKs/CIPKs) (Tuteja and Mahajan, 2007). In *Arabidopsis*, 34 different types of CDPKs have been reported, with most of them containing four EF-hand motifs within their domain structure. Some members may contain three EF-hands, e.g., CPK3/7/10/14/19/23/32; others contain variable two to three EF-hands, e.g., CPK13/25 (Cheng et al., 2002). The activity of the CDPKs largely depends upon intracellular Ca^2+^ concentration. Generally, in low intracellular calcium, the autoinhibitory domain binds with the kinase domain of the CDPKs, hence, restricting the target protein phosphorylation activity of the CDPKs. When the intracellular Ca^2+^ concentration spikes, EF hands bind with these calcium ions, and the kinase domain remains free, activating the target phosphorylation (Harmon et al., 1994; Harper et al., 1994). The full-length paralogs of barley (*Hordeum vulgare*) CDPKs, CDPK 3, and CDPK 4 were noticed to inhibit entry of powdery mildew-causing fungus *Blumeria graminis* when expressed in *Nicotiana benthamiana* (Freymark et al., 2007). The calcium sensors, in a majority of the cases, cause HR-mediated cell death by inducing ROS in the infected cells. The ectopic expression of the CaM domain was known to induce ROS in tomato protoplast by activating NADPH oxidase (Xing et al., 2001). The binding assay using host-selective toxins and non-host-selective toxins against *Alternaria solani* revealed that CDPK1 and CDPK 2 bind with them and inhibit NADPH oxidase-dependent ROS production. This demonstrates the interesting mechanism of pathogen action (Furuichi, 2020). RNA-seq analysis reveals that calcium-dependent HR response and salicylic acid were induced in pear suspension culture upon infection with an ascomycete pathogen, *Valsa pyri* (Duo et al., 2022). The induction of different classes of CDPKs was analysed by a genome-wide identification study in wild strawberries (*Fragaria vesca*) under different biotic stress factors (Xiong et al., 2022) (**Figure 1**). A total of 19 CDPKs (namely FvCDPK1 to FvCDPK19) were identified in *Fragaria vesca*, among which seven, i.e., FvCDPK1, FvCDPK4, FvCDPK7, FvCDPK15, FvCDPK17, FvCDPK18, and FvCDPK19 were upregulated upon pathogenic infection (Xiong et al., 2022).

#### 4.2.3 Calcium calmodulins

Calmodulins are small (17kDa), acidic proteins with globular subunits in the apoplast, cytosol, endoplasmic reticulum, and nucleus of plant cells (Tuteja and Mahajan, 2007). The CaM proteins contain two EF-hands in each globular domain separated by flexible α helix and are highly conserved across the plant group (Luan et al., 2002). The four EF-hands can bind four Ca^2+^, and different calmodulin sensing proteins operate downstream signalling. Calmodulin alone and activating other calmodulin-sensing proteins may instigate different physiological responses. Recently, it was observed that CaM binding protein CBP60g family were activated in response to both fungal (*Magnaporthe oryzae*) and bacterial pathogens (*Xanthomonas oryzae*). In rice there are 15 genes in CBP60 gene family, among them OsCBP60g-3, OsCBP60g-4, OsCBP60a and OsSARD-like1 were constantly upregulated (Kumari et al., 2022). In *Arabidopsis*, CBP60g plays a crucial role in immunity by directly interacting with *Arabidopsis* SYSTEMIC ACQUIRED RESISTANCE DEFICIENT 1 (SARD1) and ENHANCED DISEASE SUSCEPTIBILITY 1 (EDS1) dependent autoimmunity (Huang et al., 2021). The genome-wide analysis of the calmodulin-binding transcription activator (CAMTA) gene family was identified in Peach (*Prunus persica* L. Batsch) with varied developmental as well as stress functions (Yang et al., 2022). The CAMTA gene family is also known to induce an SA-dependent resistance pathway. It was observed that transcription activator AtSR1/CAMTA3 binds with the “CGCG box” of the NPR1 gene and activates SA-mediated pathogenesis-related (PR) protein expression. Recently it was observed that *Triticum turgidum* ssp *durum* (durum wheat) PR proteins TdPR1.2 has a CaM binding domain and are activated by TdCaM1.3 (Ghorbel et al., 2021) (**Figure 1**).

#### 4.2.4 CaM-like proteins

CMLs are another class of Ca^2+^ sensing proteins having an extra 148 amino acid sequence than CaM proteins and share minimum similarities with CaMs (Tuteja and Mahajan, 2007). CMLs are a highly unique class of sensor relay protein in plants with only 15% sequence similarities. These proteins contain two to six Ca^2+^ binding EF-hands motifs having a myriad of functions from developmental to stress response in plants (Vadassery et al., 2012). CMLs are more active against insect attack in plants; e.g., a wide array of CMLs was reported to be upregulated in soybean (*Glycine max*) in response to *Spodoptera litura* (cutworm) (Yadav et al., 2022). CML8 exhibited resistance against *Pseudomonas syringae* in *Arabidopsis thaliana* in the SA-mediated PR1 activation pathway. Although the detailed mechanism is unknown, PAMP (e.g., flg22, elf18) could not induce CML8 within *Arabidopsis*, indicating they probably induce resistance in the ETI pathway (Zhu et al., 2017). On the contrary, in tomatoes, *Solanum lycopersicum* CML 55 (SlCML55) was reported to control PR gene activation negatively, and thus, silencing lines of SlCML55 exhibited greater tolerance towards oomycetes pathogen, *Phytophthora capsici* (Zhang et al., 2022a) (**Figure 1**).

#### 4.2.5 Calcineurin B-like proteins

CBLs and CBL interacting protein kinases (CIPKs) are another essential, relatively new class of plant calcium sensors with 10 CBLs and 25 CIPKs in *Arabidopsis* (Tuteja and Mahajan, 2007). In maize, 12 CBLs genes have been identified, and most of them are reported to involve in abiotic stress tolerance. Conversely, CIPKs are comparatively more abundant in plants than CBLs. In *Lagerstroemia indica* (crape myrtle belonging to the family Lythraceae), the genome-wide analysis revealed 37 CIPKs recently (Yu et al., 2022). Although most of the functions of CBLs and CIPKs are drought, salinity, and other abiotic stress tolerance, the emerging role of these in biotic stress response is also coming up (Plasencia et al., 2021). The rice OsCIPK14 and OsCIPK15 were upregulated in response to PAMP treatment and showed resistance by activating ROS-mediated HR and cell death (Kurusu et al., 2010). On the contrary, recently, in wheat, CIPK14 was demonstrated to be negatively regulating resistance against rust fungi, *Puccinia striiformis* f. sp. *tritici* (Pst) (He et al., 2022). The *Chrysanthemum*, CmCIPK23 was observed to regulate CmTGA1 and activated nitrogen uptake during root development (Liu et al., 2022). The TGA transcription factors are also crucial for NPR1-dependent PR1 activation. The role of this class of Ca^2+^ sensors in pathogenesis needs further evaluation (**Figure 1**).

### 4.3 Ca^2+^−binding proteins without EF-hands

There are some members of calcium sensors in plants that do not possess any EF-hand motifs, e.g., phospholipase D (PLD), annexins, calreticulin, and pistil-expressed Ca^2+^ binding protein (PCP) (Tuteja and Mahajan, 2007). These calcium sensors also play pivotal roles in intracellular signalling and defence response. PLDs are highly expressed in response to pathogen attacks which hydrolyses membrane lipids to generate phosphatidic acid (PA) as a signalling intermediate. Phospholipase C (PLC), in contrast, operates in concert with PLD, where membrane lipids are hydrolysed to diacylglycerol (DAG), which produces PA by the activity of DAG kinase (Wang, 2005). Recently, the role of PLDs in symbiotic plant-microbe interaction was also revealed in some plants (Pacheco and Quinto, 2022). Annexins are another class of phospholipid-binding proteins that participate in abiotic and biotic stress response in a Ca^2+^-dependent manner (Saad et al., 2020; Gupta and Roy, 2021). The role of plant annexins in symbiotic interplay is extremely prominent. The phylogenetic and structural analysis of annexins in *Pisum sativum*-rhizobium interaction has been studied extensively (Pavlova et al., 2021). Calreticulin is the Ca^2+^ binding molecular chaperon protein involved in Ca^2+^ homoeostasis in the endoplasmic reticulum (ER). CRT1/2 and CRT3 are involved in pathogenesis signalling in *Arabidopsis thaliana* (Qiu et al., 2012). The PCPs are pistil-specific calcium sensors primarily involved in pistil growth and development. Their role in plant-microbe interaction is still largely elusive.

The above section elaborates on the sensing mechanism of intracellular Ca^2+^ signatures and the possible decoding mechanism of that signature by myriad Ca^2+^-dependent intracellular signalling transducers (**Table 1**). The Ca^2+^ oscillations in response to pathogen attack and probable defence signalling involving calcium have also been demonstrated.

## 5 The calcium and the ROS

As discussed earlier, pathogen-induced ROS production is inevitable in plant-microbe interaction. Calcium is imperative in connecting pathogen-associated signalling to ROS production and downstream defence signalling (Marcec et al., 2019). The calcium-ROS cycle is perpetuated in two cyclic events, Ca^2+^-induced ROS production (CIRP) and ROS-induced Ca^2+^ release (RICR) (Gilroy et al., 2014). The cellular ROS is principally produced by respiratory burst oxidase homologue (RBOH)/NADPH oxidase. The RBOH in plants is ubiquitously located and contains six highly conserved domains. The C terminal region contains FAD and NADPH hydrophilic domains and two heme groups, and the N terminal domain contains two Ca^2+^ binding EF-hand motifs (Chu-Puga et al., 2019). Ca^2+^ can directly bind with the EF-hand motif of the RBOH to generate CIRP in plants. In *Arabidopsis*, in response to PAMP, flg22, the receptor kinase activated botrytis induced kinase 1 (BIK1), which directly interacts with the EF-hand motifs of NADPH oxidase to release ROS in the cytosol (Wan et al., 2019). It was evident that phosphorylation in EF-hand motifs of NADPH oxidase is the prerequisite for ROS production, as kinase inhibitors significantly reduced the function (Kimura et al., 2012). The CBL1 and CBL9, along with the CIPK26, were also directly associated with the phosphorylation of EF-hands of RBOHF in *Arabidopsis* (Drerup et al., 2013). The direct role of MtCDPK5 in the phosphorylation of MtRbohB, MtRbohC, and MtRbohD to generate ROS in response to pathogenesis has also been demonstrated (Yu et al., 2018). Recently, genome-wide analysis of CDPK genes has revealed different CDPK-RBOH clusters in response to chilling stress in Peach (Zhao et al., 2022); their details role in pathogenic stress tolerance needs to be further clarified.

RICR is mediated by the direct action of ROS on the hyperpolarisation of Ca^2+^ channels (Gilroy et al., 2014). Stelar K^+^ outward rectifier (SKOR) channel and Ca^2+^ sensitive annexins were found to be directly influenced by the ROS (Garcia-Mata et al., 2010; Richards et al., 2014). The annexin-induced Ca^2+^ elevation in response to ROS is mediated by extracellular nucleotides (eATP or eADP). The first reported eATP receptor *Arabidopsis thaliana* DORN1 (Which does Not Respond to Nucleotides), coordinated the ROS-induced Ca^2+^ balance in plants (Mohammad-Sidik et al., 2021) (**Figure 1**). Hence, ROS may also act as the stress marker for calcium signalling. In *Arabidopsis thaliana*, H_2_O_2_-INDUCED CA^2+^ INCREASES 1 (HPCA1) may act as prominent markers for ROS-induced Ca^2+^ signalling. Similarly, Sucrose-non-fermenting-1-related Protein Kinase 2.6/OPEN STOMATA 1 (OST1) is required for the cell-to-cell transition of ROS (Fichman et al., 2022).

The above section summarises interesting signalling perpetuation between Ca^2+^ accumulation and ROS production. The cyclic events of CIRP and RICR in response to pathogenesis have been demonstrated (**Table 1**).

## 6 MAPK signalling cascade and Ca^2+^ signal overlap

CDPKs and MAPKs are both very much crucial for the defence signalling pathway. Pathogen-induced intracellular ROS and Ca^2+^ signatures can induce calcium sensors and MAPK signalling cascades. The parallel induction of these two pathways has raised the question, is these two pathways independently operating or have some common players? In animal pathophysiology, cross-talk between these two pathways was evident in some cases, but in the case of plants, the reports are intangible. If it has been analysed minutely, the C terminal end of most of the CDPKs is highly conserved, and only the N terminal end is variable containing N-myristoylation and palmitoylation. In *Arabidopsis*, 27 out of 34 CDPKs showed these sites in the second position of their amino acid series. These structures are required for subcellular localisation and membrane attachment. A similar structure was also observed in other Ca^2+^ sensors, e.g., CBLs and CIPKs. Whereas MAPKs were largely devoid of such structure. Only four MAPKs out of 20 in *Arabidopsis* showed N-myristoylation sites (Wurzinger et al., 2011). The MAPKs are mostly cytosolic and influenced by different secondary messengers. The membrane phosphatases were reported to activate MAPKs in response to a myriad of developmental cues. Protein phosphatases may be operative due to the possible signalling overlap between CDPKs and MAPKs. The integrated action of phosphatases and protein kinases (CDPKs, MAPKs) in plant immunity is an emerging field of study in plant immunology. The PAMP flg22, elf18, or chitin interacted with PRRs fls2, EFR/BAK1 or LYK5/CERK1 simultaneously, which further activates BIK1 integrating ROS mediated calcium signalling on the one hand and MAPK signalling cascade on the other hand (Erickson et al., 2022). The ectopic expression of truncated *Nicotiana tabacum* CDPK2 lacking its regulatory autoinhibitory domain and calcium binding domain can induce ROS-mediated calcium signalling and inhibits MAPK-mediated stress signalling (Ludwig et al., 2005). Although further insights are required, current understanding indicates the overlap between Ca^2+^-dependent kinase-MAPK pathways in controlling plant-microbe interaction.

This section demonstrates a fascinating and emerging field of plant immunology. The two most crucial signalling cascades in plants, CDPKs, and MAPKs, may overlap in their intracellular signal transduction (**Table 1**). Further in-depth studies have been urgently necessitated to deliver more insight into this matter.

**Table 1:** **A** list of plant-microbe interaction studies revealing calcium signalling plays a crucial role.

Sl. No.	Name of the plant	Name of the pathogen	Methods of study	Disease/interaction	Signalling pathways modulated	References
1	*Medicago truncatula*	Rhizobium	Knock out mutation	Nodulation/Symbiosis	RBOHs, CDPKs etc.	(Yu et al., 2018)
2	Strawberry	*Botrytis cinerea*	RNA Seq transcriptomics	Gray mould disease	CDPKs and MAPKs	(Xiong et al., 2018)
3	*Arabidopsis thaliana*	*Sclerotinia sclerotiorum* (Lib.)	Quantitative disease resistance (QDR) response		CNGCs, CDPKs, CaM, CaMK, CRKs etc.	(Wang et al., 2019)
4	*Vitis vinifera*	*Lasiodiplodia theobromae*	Dual RNA Seq		CDPKs, LRR, LRKs etc.	(Gonçalves et al., 2019)
5	*Gossypium hirsutum*	*Begomoviruses*		Cotton leaf curl Multan beta satellite (CLCuMB)	Calcium signalling and Gh-CML11	(Kamal et al., 2019)
6	*Triticum aestivum* L.	*Rhizoctonia cerealis*	Transcription assays, Virus-induced gene silencing (ViGS), subcellular localization	Sharp eyespot	TaCML36, Chitinase 1, PDF35, PR17C, the ethylene response factor etc.	(Lu et al., 2019)
7	*Crop plants*	*Begomovirus*	Genome wide			(Gnanasekaran et al., 2019)
8	*Citrus sinensis*	Arbuscular mycorrhizal fungi (AMF)	Genome-wide identification and expression analysis (GWIEA)	Association	CDPKs	(Shu et al., 2020)
9	*Leifsonia xyli* subsp. *xyli* (Lxx)	*Saccharum officinarum L.*	Transcriptomics	Sugarcane ratoon stunting disease (RSD)	CDPKs, Zinc finger proteins, NBS LRR etc.	(Zhu et al., 2021)
10	*Lens culinaris* Medik.	*Rhizoctonia bataticola*	RNA-Seq	Dry root rot	CDPKs, CaMKs, LRR-RLKs, ROS, MAPKs, SA/JA etc.	(Mishra et al., 2021)
11	*Panicum miliaceum* L.	*Sporisorium destruens*	RNA-Seq	Smut disease	CDPKs. And calcium signalling	(Jin et al., 2021)
12	*Nicotiana tabacum*	*Phytophthora nicotianae*	Comparative transcriptome (RNA Seq)	Root rot, crown rot, fruit rot, and leaf and stem infections	RLP/RLK, CNGC, CDPKs, MAPKs etc.	(Meng et al., 2021)
.13	*Medicago sativa* L.	*Fusarium proliferatum L1*	RNA seq	Root rot	CDPKs, CIPKs, ROS etc.	(Zhang et al., 2022b)
14	Apple (*Malus* sp.)	*Valsa mali*	Transcriptomics	Valsa canker	CNGC and CDPKs	(Wang et al., 2022)
15	*Arabidopsis thaliana*	PAMPs	Plant-microbe interaction	PTI	CAM-BINDING PROTEIN 60-LIKE G (CBP60g), CALCIUM-DEPENDENT PROTEIN KINASE5 (CPK5), TOUCH 3 (TCH3) CALMODULIN (CAM) 1/4/6 and CPK4/5/6/11 etc.	(Sun et al., 2022a)
16	*Poa pratensis* L.	*Blumeria graminis* (DC.) Speer	RNA-Seq	Powdery mildew	Glutamine synthetase, CDPKs etc.	(Sun et al., 2022b)
17	*Arabidopsis thaliana*	*pathogen*		Biotic stress	Calcium-CaM-AtSR1 interaction module.	(Yuan et al., 2022)
18	*Arabidopsis thaliana*	*Pseudomonas syringae* and *Botrytis cinerea*	Loss of function mutation and transient overexpression.	Blast and rot	Calcium signalling and MAPK signalling	(Bai et al., 2022)
19	*Citrus*	*Penicillium digitatum and P. italicum*		Favoured fungal growth, sporulation, virulence, and environmental stress tolerance of the pathogen.	PiCaMK1 (CaMK of the pathogen)	(Li et al., 2022)
20	*Oryza sativa*	*Xanthomonas oryzae* pv. *oryzicola* (Xoc)	Transcriptomics (RNA Seq)	Bacterial leaf streak	Ethylene, JA, SA, MAPK and calcium signalling.	(Tang et al., 2023)
21		Plant-microbe or plant symbiotic interaction		Pathogenesis/symbiosis	CDPKs, CIPKs, CaMKs, RBOHs, ROS signalling, salicylic acid (SA), pathogenesis-related protein 1 (PR1), and negative regulation of Ca^2+^ signalling.	(Yuan et al., 2017)
22	*Arabidopsis* and *Nicotiana benthamiana* etc.	Plant-microbe or plant symbiotic interaction		Pathogenesis/symbiosis	The role of calcium signalling in plant-microbe interplay and plant developmental events.	(Evangelisti et al., 2014)
23		Fungal and microbial pathogens			RLKs and CDPKs	(Barka et al., 2023)
24		Plant biotic interactions		Plant-microbe interaction, beneficial or harmful	CaMs, CMLs, CDPKs etc., in immunity, mutualism; positive and negative regulation of plant immunity.	(Aldon et al., 2018)
25	*Medicago* and other leguminous plants	Plant -Rhizobium interaction		Symbiosis	Interplay between CNGC15a, b, c, DMI1, Ca^2+^-ATPase 8, CCaMK and NOD factors. The regulatory mechanism of CaM-CCaMK-DELLA-CYCLOPS complex.	(Yuan et al., 2022)
26		Rhizobium-legume symbiosis (RLS) and arbuscular mycorrhizas (AM)		Plant-microbe interaction	Common symbiotic pathway (CSP) regulating CCaMK, MCA8 calcium as an intermediate signalling ion. It also incorporates CYCLOPS as a common substrate.	(Genre and Russo, 2016)

## 7 Conclusion and future question

The plant-microbe interplay is classically distinguished into two phases, PTI and ETI. There are many arguments regarding the distinct partitioning of these immunogenic events in plants, as a clear distinction is absent between PTI and ETI. Among many other inevitable events in response to biotic ingression, perturbations of calcium concentration in the cellular milieu and calcium-induced signalling are paramount. The paradigm shift of calcium concentration in cell cytosol from “resting concentration” is specific to pathogens as well as conditions of the infection. This specific calcium concentration in response to specific stress is defined as a “calcium signature”. Several calcium sensors in plants carry out the exciting phenomenon of decoding these calcium signatures. The CDPKs are the major players in calcium-mediated signalling in plants. The ROS is in integrated association with calcium signalling as “calcium-induced oxidative burst” and “ROS-induced calcium influx” is well documented in plant immunity. The study regarding integrating other signalling pathways with calcium signalling is sparse. MAPK signalling is another vital signalling cascade in plants against biotic ingression. Although the indication of the overlap between MAPK cascade and calcium signal is there, more work is still needed to solve this signalling jigsaw. Detecting the precise cytosolic and organellar concentration of calcium and decoding the same in response to specific pathogen attacks is another urgent need to develop an ionic calcium map in plants. Alternatively, more focus should be given to the interconnection of calcium signalling with other signalling pathways. Bridging the gap between organellar “calcium signature” and overlapping signal transduction pathway in future research may bring forth useful information to develop sustainable resistance in crop plants. Along with the positive roles of Ca^2+^ in plant-microbe interaction, more focus on the opposing roles of the same on plant immunity may help fill up the lacunae in understanding the calcium signalling cascade in plants upon different stress factors and developmental cues.

## Author contributions

AB, AR conceptualized the study. AB wrote the first draft. AR, AC and AB prepared the final draft. All authors contributed to the article and approved the submitted version.

## Supplementary material

Supplementary Excel 1 The details of interacting partners after STRING (version 11.5) analysis using *Arabidopsis* CDPK1, CaMK4, CAM8 and CBL1.

Supplementary Excel 2 The interaction nodes of CDPK1 and CBL1 as found in the co-expression analysis using STRING (version 11.5).

Supplementary Figure 1 Co-expression analysis of CDPK, CaMK, CBL, CML observed in *Arabidopsis thaliana* using STRING (version 11.5).
