# Supplementary figures and images for: The captivating role of calcium in plant-microbe interaction
Source: Front Plant Sci. 2023 Mar 3;14:1138252. doi: 10.3389/fpls.2023.1138252 (PMC10020633; doi:10.3389/fpls.2023.1138252)

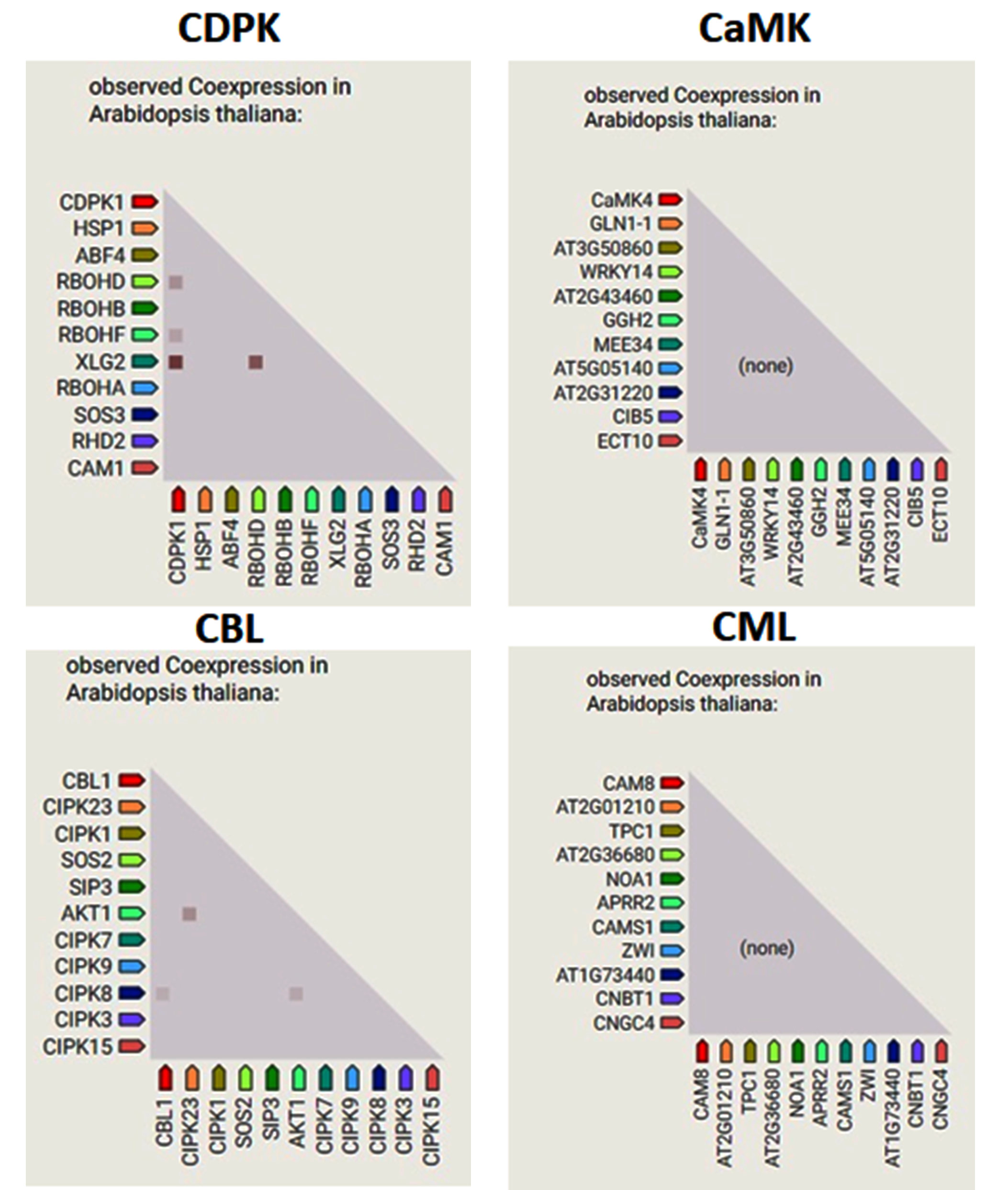

Supplement: Supplementary Figure 1 — Co-expression analysis of CDPK, CaMK, CBL, CML observed in Arabidopsis thaliana using STRING (version 11.5). [file Image_1.tif]
